# Recovery of Neodymium (III) from Aqueous Phase by Chitosan-Manganese-Ferrite Magnetic Beads

**DOI:** 10.3390/nano10061204

**Published:** 2020-06-19

**Authors:** Sergio Valverde Durán, Byron Lapo, Miguel Meneses, Ana María Sastre

**Affiliations:** 1 School of Biochemistry and Pharmacy, Universidad Técnica de Machala, FCQS, BIOeng Group, 070151 Machala, Ecuador; svalverde_est@utmachala.edu.ec; 2 Department of Chemistry, Universidad Técnica Particular de Loja, San Cayetano alto, 110150 Loja, Ecuador; mameneses@utpl.edu.ec; 3 Department of Chemical Engineering, Universitat Politècnica de Catalunya, EPSEVG, Av. Víctor Balaguer 1, 08800 Vilanova i la Geltrú, Spain; 4 School of Chemical Engineering, Universidad Técnica de Machala, FCQS, BIOeng Group, 070151 Machala, Ecuador; 5 Department of Chemical Engineering, Universitat Politècnica de Catalunya, ETSEIB, Diagonal 647, 08028 Barcelona, Spain; ana.maria.sastre@upc.edu

**Keywords:** rare earth, adsorption, magnetic, spinel ferrite, MnFe_2_O_4_

## Abstract

Neodymium is a key rare-earth element applied to modern devices. The purpose of this study is the development of a hybrid biomaterial based on chitosan (CS) and manganese ferrite (MF) for the recovery of Nd(III) ions from the aqueous phase. The preparation of the beads was performed in two stages; first, MF particles were obtained by the assessment of three temperatures during the co-precipitation synthesis, and the best nano-MF crystallites were incorporated into CS to obtain the hybrid composite material (CS-MF). The materials were characterized by FTIR, XRD, magnetization measurements, and SEM-EDX. The adsorption experiments included pH study, equilibrium study, kinetics study, and sorption–desorption reusability tests. The results showed that for MF synthesis, 60 °C is an appropriate temperature to obtain MF crystals of ~30 nm with suitable magnetic properties. The final magnetic CS-MF beads perform maximum adsorption at pH 4 with a maximum adsorption capacity of 44.29 mg/g. Moreover, the material can be used for up to four adsorption–desorption cycles. The incorporation of MF improves the sorption capacity of the neat chitosan. Additionally, the magnetic properties enable its easy separation from aqueous solutions for further use. The material obtained represents an enhanced magnetic hybrid adsorbent that can be applied to recover Nd(III) from aqueous solutions.

## 1. Introduction

Rare-earth elements (REEs) are gaining attention in technological areas because of their properties and applications [1]. Among REEs, neodymium is a key element in the technological industry, which is mainly applied in magnets and electric motors [2], and it is considered to be a critical element due to its possible scarcity in the future. Moreover, some recent studies reveal the introduction of Nd(III) in water streams [3]. Thus, the development of technologies toward the recovery or removal of Nd(III) is highly relevant.

The recovery of Nd(III) from the aqueous phase is possible through the use of several technologies such as fractional precipitation, ion-exchange, or solvent extraction after leaching the solids (i.e., from primary sources or end-of-life products). However, these processes present some disadvantages. For instance, ion-exchange is used mainly in low-scale processes and is mostly used for heavy REEs [4], and solvent extraction requires high quantities of organic solvents and big infrastructures [5]. As an alternative, adsorption and, particularly, biosorption has an enormous potential. The special attention to biosorption is because of its low cost and use of sustainable materials [6].

Chitosan is one of the most abundant biopolymers in the earth and is considered a relevant material used in adsorption of metals and REEs [7]. On the other hand, spinel ferrites (SF) are inorganic magnetic particles that present excellent adsorption capacities for metal removal [8]. The main advantage of this over other forms of magnetic iron particles such as Fe_3_O_4_, α-Fe_2_O_3_, γ-Fe_2_O_3_, and zero-valent iron is their enhanced physical–chemical stability under harsh environmental conditions, which avoid the particles’ aggregation, changes in their oxide form, and conservation of their magnetic properties [9].

Chitosan (CS) and SF can be blended to form hybrid biomaterials. The development of these kinds of materials (biopolymer and inorganic particles) is important particularly because the hybrid structures can be tuned to obtain suitable properties toward the desired applications [10]. As well as this, the biopolymers can act as a matrix for inorganic particle immobilization (micro- or nano-sized). Recent studies had demonstrated the capability of CS-SF composites for the adsorption of heavy metals such as Cu(II), Cr(VI), Hg(II), and Pb(II) [11,12,13]; however, there is no scientific literature related to the removal of Nd(III) ions or any other rare-earth element, and only a handful of papers have investigated the use of CS-magnetic (different forms than SF) for Dy(III), Nd(III), Er(III), Yb(III), and La(III) [14,15,16,17].

Sustainable processes also require competitive costs of production; therefore, the implementation of effective and low-cost manufacture methods is crucial for their introduction into the industry. There are various methods of obtaining spinel ferrite, including controlled oxidation, thermic decomposition, and sol–gel and co-precipitation methods [18]. While controlled oxidation requires an inert atmosphere [18], thermic decomposition and sol–gel are relatively simple but need high temperatures [19]. Among the cited methods, co-precipitation represents the most simple and low-cost technology, due to the simple process and low temperatures (<100 °C) for the synthesis with acceptable magnetic properties.

This research is focusing on the development of a hybrid material in the form of beads, based on chitosan and MnFe_2_O_4_, for the recovery of Nd(III). The study was divided in two stages. Firstly, particles of MnFe_2_O_4_ were obtained, using three temperature levels in the co-precipitation method. Secondly, gel beads of CS-manganese ferrite (MF) were formed by the incorporation of the best MF into CS, and the technological aspects toward the Nd(III) recovery in the aqueous phase were studied. The developed bio-beads represent an improvement in the preceding studies carried out by our research group [20,21,22], as well as filling a gap in the scientific knowledge regarding the use of CS-MF material for Nd(III) recovery in aqueous adsorption.

## 2. Materials and Methods

### 2.1. Chemicals

Neodymium nitrate hexahydrate (Nd(NO_3_)·6H_2_O Sigma-Aldrich, St Louis, MO, USA), chitosan (acetylation degree of chitin 0.13, Sigma-Aldrich, St Louis, MO, USA), manganese(II) sulphate (MnSO_4_·H_2_O Sigma-Aldrich, St Louis, MO, USA), acetic acid (CH_3_COOH, 99.7%, 6H_2_O Sigma-Aldrich, St Louis, MO, USA), hydrochloric acid (HCl, 37.4%, J.T. Baker, Phillipsburg, NJ, USA), sodium hydroxide (NaOH, 97%, Panreac, Barcelona, Spain), nitric acid (HNO_3_, 69.0%, J.T. Baker, Radnor, PA, USA), sodium chloride (NaCl, 99.5%, Prolabo, Fontenay-sous-Bois CEDEX, France), iron(III) chloride (FeCl_3_·6H_2_O, 99–102%, Sigma-Aldrich, St Louis, MO, USA), ethylenediaminetetraacetic acid disodium salt dihydrate (EDTA, 99% Panreac, Barcelona, Spain), methanol (98% Panreac), ethanol (etOH, 96% Panreac), methanol (meOH, UV-IR-HPLC isocratic, Panreac), and deionized water type II laboratory water were used.

### 2.2. Synthesis of Manganese-Ferrite

The coprecipitation method was used to obtain the MnFe_2_O_4_ particles, in which the effect of the temperature in the synthesis of the material was evaluated. The temperature of each procedure was varied at 60, 70, and 80 °C, and the resultant materials were labeled as MF-60, MF-70, and MF-80, corresponding to 60, 70, and 80 °C, respectively. To prepare the material, two solutions of FeCl_3_·6H_2_O 0.1 M and MnSO_4_·H_2_O 0.05 M were mixed in the stoichiometric relationship Fe:Mn of 2:1; then, 9 mL of NaOH 2M was swiftly added to reach the exact pH of 10.5. The solutions had been previously heated at the corresponding experimental temperature (60, 70, or 80 °C) and agitation was kept at 500 rpm for 120 min. Lastly, the obtained particles were washed several times with deionized water and totally dried in an air convection oven (Barnstead Thermolyne-Cimarec) for 48 h at 80 °C.

### 2.3. Synthesis of Chitosan-Manganese Ferrite Magnetic Beads

Chitosan without prior pre-treatment was dissolved in 40 mL of acetic acid 0.5 M (2.5% *w*/*v*), and was blended at 500 rpm for 60 min at 30 °C. This solution was added with 0.5 g of MF-60 and 20 mL of acetic acid 0.5 M, and newly agitated at 1200 rpm for 120 min at 30 °C. The final mass relationship of CS:MF-60 was 2:1.

For the bead formation, the previously prepared suspension was pumped with a peristaltic pump (Pharmacia LkB Pump) at 3 mL/min and added drop by drop in a NaOH 4 M solution. The coagulation process was kept for 48 h at 10 °C. Prior to the application in adsorption studies, the beads were washed with deionized water to remove the excess of NaOH and dried in an air convection oven for 12 h at 40 °C. The resultant beads were labeled as CS-MF.

### 2.4. Characterization

Infrared spectra were collected from 450 to 4000 cm^−1^ in a FTIR-ATR Thermo Scientific Nicolet 6700 (Madison, WI, USA). Magnetization hysteresis curves of the magnetic materials were obtained at 300 K in a superconducting quantum interference device (SQUID, Quantum Design magnetometer, Darmstadt, Germany). XRD spectra patterns under Cu Kα radiation from 4 to 100° 2θ, and an exploratory velocity of 0.02 °/s, were obtained with a Bruker D8 Advance (Bruker AXS GmBH, Karlsruhe, Germany). The pH of zero charge potential (pH_pzc_) was evaluated according to previously published work of [21]. The surface morphology and elemental distribution before and after the sorption experiments of the resultant beads were determined in a scanning electron microscope with an energy-dispersive X-ray probe (SEM-EDX-Phenom XL, Rotterdam, The Netherlands). Particle size distribution measurements were performed in a Zetasizer Nano Z (Malvern Panalytical Ltd., Malvern, UK).

### 2.5. pH Study

The effect of pH in the adsorption uptake of Nd(III) was evaluated in both MF particles and the CS-MF beads. The pH was varied at 2.0, 4.0, and 6.0. Solutions of 25 mL of 50 mg/L of Nd(III) concentration were added with 25 mg of the sorbent material (i.e., sorbent dosage (SD) of 1 g/L). The pH of the solutions was carefully adjusted by the adding of NaOH or HNO_3_. The initial (pH_i_) and final pH (pH_e_) were recorded. Agitation speed (AS) and contact time (CT) were fixed at 150 rpm and 24 h respectively. The Nd(III) analysis was measured in an ICP-OES (Perkin Elmer Optima 7300). Equation (1) was applied to calculate the sorption uptake or sorption capacity of the materials.

Sorption uptake equation:

(1) $$q_{e}=\frac{V(C_{i}-C_{e})}{w}$$

where q_e_ is the adsorption capacity in (mg/g), C_i_ and C_e_ are the initial and equilibrium concentrations, respectively, V is the volume in L, and w is the mass of sorbent added expressed in grams. The experiments were carried out in triplicate.

### 2.6. Equilibrium Study

Solutions of 25 mL of C_i_ from 5 to 200 mg/L of Nd(III) were added with 25 mg of sorbent materials (SD: 1 g/L) and agitated during 24 h at 150 rpm in a laboratory orbital shaker. The pH of the solution was adjusted to 4.0, which was set as the optimum pH.

Langmuir, Freundlich, and Sips nonlinear models were used to fit the equilibrium data obtained, according to Equations (2)–(4), respectively [23].

Langmuir equation:

(2) $$q_{e}=\frac{q_{\max}{\mathrm{bC}}_{e}}{1+{\mathrm{bC}}_{e}}$$

Freundlich equation:

(3) $$q_{e}=K_{F}C_{e}^{1/n}$$

Sips equation:

(4) $$q_{e}=\frac{q_{\max}K_{s}C_{e}^{1/\mathrm{ms}}}{1+K_{s}C_{e}^{1/\mathrm{ms}}}$$

where q_e_ is the adsorption capacity calculated by Equation (1) (mg/g), C_e_ is the equilibrium concentration (mg/L), q_max_ is the Langmuir or Sips maximum capacity in the monolayer expressed in (mg/g), b is the Langmuir constant in (L/g), K_F_ is the Freundlich constant, n is the sorption intensity, K_s_ is the Sips equilibrium constant in (L/mg), and ms is the Sips model exponent. The experiments were carried out in triplicate.

### 2.7. Kinetics

In 1 L of Nd(III) solution of Ci 50 mg/L and pH 4.0, 100 mg of CS-MF (SD: 1 g/L) was added, and several samples were then taken through time to measure the remaining Nd(III) concentration. Pseudo-first-order (PFORE), pseudo-second-order (PSORE), Elovich, and Weber and Morris models were assessed to fit the experimental data and obtain the kinetics parameter according to Equations (5)–(8):

Pseudo-first-order equation (PFORE):

(5) $$\frac{{\mathrm{dq}}_{t}}{\mathrm{dt}}=K_{1}\left(q_{e}-q_{t}\right)$$

Pseudo-second-order equation (PSORE):

(6) $$\frac{{\mathrm{dq}}_{t}}{{\left(q_{e}-q_{t}\right)}^{2}}=K_{2}\mathrm{dt}$$

Elovich equation:

(7) $$(\frac{{\mathrm{dq}}_{t}}{d_{t}}=\alpha \exp\left(-{\beta q}_{t}\right)$$

Weber and Morris equation:

(8) $$q_{t}=K_{\mathrm{int}}t^{1/2}+C$$

where q_e_ is the equilibrium sorption capacity (mg/g), q_t_ is the sorption capacity (mg/g) at any time t (h), k_1_ is the PFORE rate constant (1/min), k_2_ is the PSORE rate constant (g/mg min), α is the initial adsorption rate (mg/g min), β is a desorption constant related to the extent of surface coverage and activation energy for chemisorption, k_int_ is the intraparticle diffusion rate constant in mg/g*min^1/2^, and C is the initial adsorption (mg/g).

### 2.8. Desorption Cycles

Sorption-desorption cycles were carried out in two stages; first, to choose a proper eluent, one sorption-desorption cycle using HCl (pH 3.5), EDTA (0.05 M), ethanol (96% *v*/*v*), and methanol (98%) was used to desorb the REEs. Secondly, the best eluent, in terms of the recovery percentage desorbed, was applied in several sorption–desorption experiments.

(9) $$\mathrm{Recovery}\;\%=\frac{C_{D}{*V}_{D}}{(C_{i}-C_{e}){*V}_{A}}*100$$

where C_D_ and V_D_ are the concentration (mg/L) of REEs and the volume (L) in the eluted solution experiments (desorbed), respectively, C_i_ and C_e_ are the initial and equilibrium concentration (mg/L), respectively and V_A_ is the volume (L) used for adsorption experiments. The experiments were carried out in triplicate.

## 3. Results

### 3.1. MnFe_2_O_4_ Particles

#### 3.1.1. Characterization

**Figure 1:**
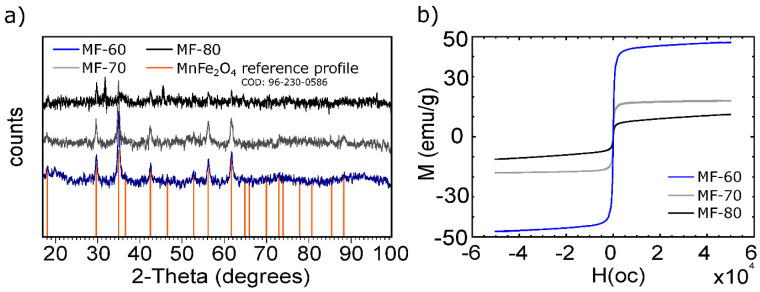
MnFe_2_O_4_ characterization. (**a**) XRD patterns, (**b**) magnetic hysteresis loops (300 K).

The formation of MnFe_2_O_4_ was confirmed by XRD analysis (Figure 1a), and the three materials presented a majority phase at (34.96) [24]. However, MF-60 matches by 70% with MnFe_2_O_4_, according to the crystallography open database (COD): 96-230-0586. The peaks at (42.43), (56.14), and (61.74) are characteristics of a spinel structure. MF-70 and MF-80 present similarity between them, with the appearance of a peak at (61.89) in MF-80. The crystallite size was determined with the Scherrer equation, which corresponds to 30.3, 36.5, and 40.8 nm.

The magnetic properties in the three spinel-ferrites were determined; the magnetic hysteresis loops are depicted in Figure 1b. The typical behavior of ferromagnetic materials is observed. MF-60 particles have the most significant saturation magnetization (Ms), reaching 49.6 emu/g, while MF-70 and MF-80 reach 17.2 and 11.3 emu/g, respectively. The high Ms of MF-60 confirms the largest formation of MnFe_2_O_4_, which is in concordance with the XRD patterns and lower crystallite size, while lower magnetizations in MF-70 and MF-80 confirm the partial formation of MnFe_2_O_4_.

Thus, using this synthesis procedure, 60 °C is a suitable temperature to form the chemical spinel structure with a saturation magnetization and crystallite size suitable for the adsorption purposes. Other authors such as [8], using the same co-precipitation method at 80 °C, obtained MF with Ms of around 20 emu/g. Besides, [24] reported a slightly better Ms of 66 emu/g for MF synthetized by the one-step microwave hydrothermal method at 120 °C.

#### 3.1.2. pH Dependence

**Figure 2:**
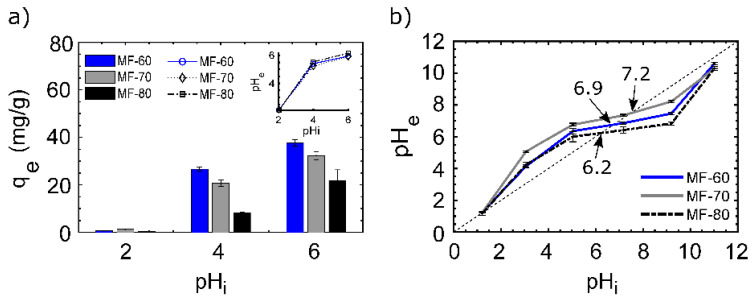
pH dependence: (**a**) pH vs. adsorption uptake, (**b**) pH_pzc_ of spinel-ferrites. Conditions: (**a**) T: 25 °C, sorbent dosage (SD): 1 g/L; AS: 150 rpm; CT: 24 h; initial concentration (Ci): 50 mg/L; (**b**) T: 25 °C, SD: 1 g/L; AS: 150 rpm; CT: 24 h; electrolyte: NaCl 0.01 M.

pH is one of the most important factors that influence the adsorption process [20]. In Figure 2a, the influence of pH in the recovery of Nd(III) by MF particles is depicted. The effect of the pH was studied at pHs between 2 to 6, particularly because at pH > 6, the precipitation of Nd(III) in its insoluble hydroxide form (Supplementary Figure S1) is produced.

The uptake capacity (q_e_) was higher as the pH was increased. It is notable that MF-60 achieves the highest performance in contrast to MF-70 and MF-80. Overall, for the three MFs, acid conditions (i.e., pH 2) did not favor the adsorption process, due to the fact that high proton concentration affects the metal-sorbent interactions, reducing the capability of Nd(III) ions for binding active sites, while, as long as the pH is increased, the adsorption is favored, reaching the maximum adsorption capacity at pH 6 (q_e_ = 37.87 for MF-60), representing the 75.75% of efficiency.

The trend in the differences of adsorption efficiency could be explained by the role of the pH_pzc_, which is defined as the value in which the total charge (external and internal) of the material is neutral [25], and this property is used for determining the affinity of an adsorbent for a specific sorbate [26]. The pH_pzc_ (Figure 2b) of MFs shows that the materials present a positive surface at the experimental conditions (pH < pH_pzc_) [27]. The MF particles pH_pzc_ were determined at 6.9, 7.2, and 6.2 for MF-60, MF-70, and MF-80, respectively. According to [28], at pHs under pH_pzc_, it is possible a protonation of active sites, as well as cations, can compete for the same active sites, and as the pH reaches the pH_pzc_, the interactions between the Nd(III) and active surface sites are major, because there are less available protons.

On the other hand, the differences in the performance at the same pH are very notable (Figure 2a). MF-60 particles uptake 22% more Nd(III) than MF-70 and 3 times more than MF-80 at pH 4. Similarly, at pH 6, MF-60 particles adsorb 14% and 42% more than MF-70 and MF-80, respectively. This behavior is attributed to the crystallite size and purity of the particles, which were higher for MF-60 > MF-70 > MF > 80, in line with the adsorption behavior. These material features provide high particle size, surface area, and a larger number of active surface sites (corners, edges, steps), as well as hydroxyl groups, to facilitate and improve the metal adsorption [29,30].

Many magnetic particles based on MFs have been tested for Nd(III) and heavy metal removal from aqueous solutions. Table 1 shows various results with their related pH and experimental uptake capacity (q_e_). It is noted that MF-60 particles present a competitive performance for Nd(III) recovery against magnetite particles. Besides, MFs applied to the removal of Pb and Cr showed capacities under the reported MF uptake.

### 3.2. CS-MF Beads

After the evaluation of the MFs particles, MF-60 was selected to be incorporated into the CS. The CS-MF material was manufactured in the form of beads as this spherical form could be suitable for use in batch or column adsorption systems. Moreover, in this format, the MF microparticles can be fixed into the chitosan for the enhancement of its adsorption capabilities and preventing the presence of microparticles in the final aqueous phase.

#### 3.2.1. Morphology and Elemental Characterization

**Figure 3:**
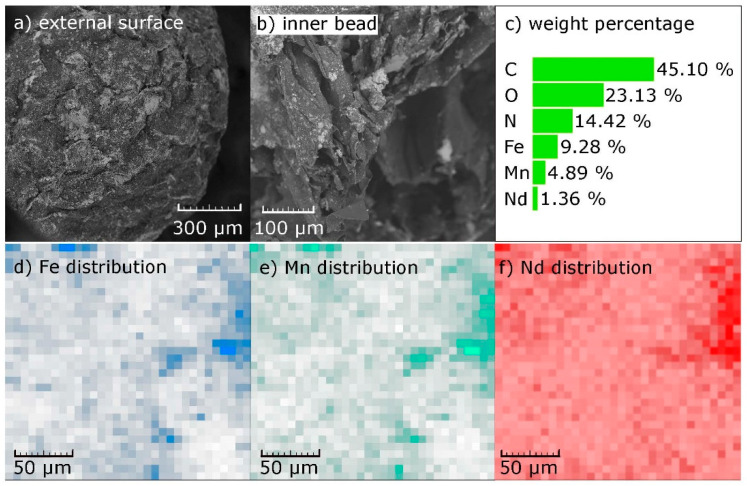
SEM-EDX analysis of chitosan-manganese ferrite (CS-MF) beads. SEM images of (**a**) the external bead surface, (**b**) the axial view of the beads, (**c**) EDX analysis result of bead after Nd(III) adsorption, (**d**–**f**) EDX elemental distribution of Fe, Mn, Nd, respectively after Nd(III) adsorption.

The morphology observations and the elemental distribution of the beads after Nd(III) uptake are shown in Figure 3. On the external bead surface (Figure 3a), roughness and cracked features are observed, which helps the beads enter into the aqueous phase and, consequently, the transport of Nd(III) ions into the CS-MF material. The axial view of the beads (Figure 3b) presents a high roughness and macro porosity of about 80–300 µm, which are more pronounced than in the external surface.

The EDX analysis reveals the presence of C, O, N, Fe, Mn, and Nd on the surface of the bead after Nd(III) adsorption (Figure 3c). The C, O, and N are related to the CS matrix, while Fe and Mn are related to the incorporated MF, which were homogeneously distributed along the CS (Figure 3d,e). Furthermore, in Figure 3f, the homogeneous Nd distribution after adsorption is observed.

#### 3.2.2. FTIR, XRD, and Magnetic Evaluation

**Figure 4:**
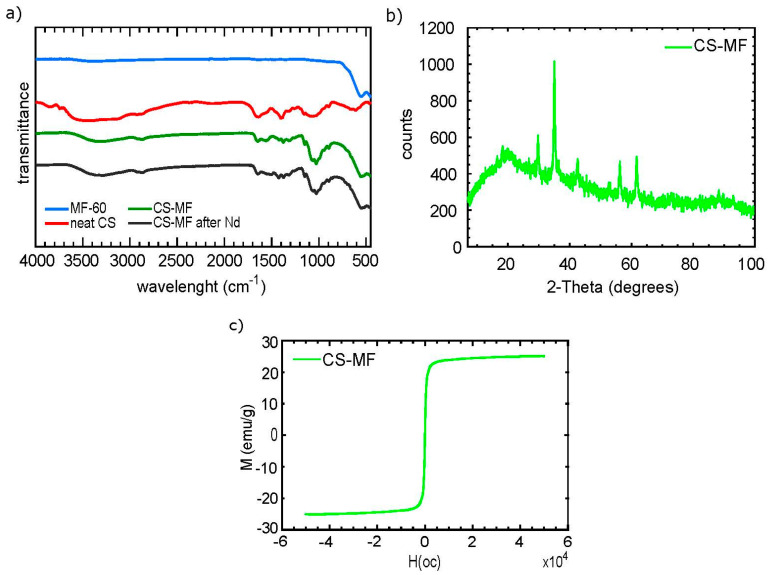
CS-MF characterization. (**a**) FTIR spectra, (**b**) XRD spectrum, (**c**) magnetic hysteresis loop.

The FTIR spectra (Figure 4a) show the functional groups present on the material surfaces of CS-MF, MF-60, and neat CS. The spectra of MF-60 clearly indicate the peak at 552 cm^−1^ related to the Fe-O vibration, which is indicative of the spinel manganese-ferrite structure [34]. CS and MF-60 FTIR spectra before the adsorption showed some common signals, i.e., a peak at 3281 cm^−1^, attributed to the hydroxyl-related groups (stretching of C-OH and Fe-OH) and the stretching vibration of the primary amine of the N-H group of CS [35], and a band at 2885 cm^−1^, which is related to the symmetric groups of -CH_2_ [36]. In addition, the signal at 1640 cm^−1^ is attributed to the C=O stretching vibration related to the carboxylates present in polysaccharides [35], and the peaks at 1374 and 1027 cm^−1^ are attributed to the C-O-C and C-O of CS, respectively [16]. Despite the similitudes between CS-MF and CS, a marked difference was identified in CS-MF at 552 cm^−1^, which is attributed to the incorporation of MnFe_2_O_4_ into CS-MF. The incorporation of Nd(III) after the adsorption was observed by the identification of two shifts in the IR spectra, one from 1554 to 1514 cm^−1^ related to the C=O stretching in secondary amide [37], and a second from 1418 to 1429 cm^−1^ related to the amide II groups of chitosan [38].

The XRD pattern of CS-MF (Figure 4b) confirms the incorporation of MF into CS; although the XRD pattern is mostly similar to the MF (Figure 1a), there are some differences. For instance, the peak at 61.74 disappears in CS-MF, probably due to the CS interaction with MF; besides, a low decrease between the atomic planes (A) was observed. Again, the database (COD: 96-230-0586) agrees by 57% with MF, and this is due to the presence of CS in the CS-MF beads.

The magnetic hysteresis loop of CS-MF (Figure 4c) indicates an Ms of 21.4 emu/g, which is less than that of its MF-60 neat particles, but higher than those of the MF-70 and MF-80 neat particles (Figure 1b). The drop in the Ms is expected, because of the presence of CS; however, this value is enough to be considered feasible to be separated by magnetic methods.

#### 3.2.3. pH Dependence

**Figure 5:**
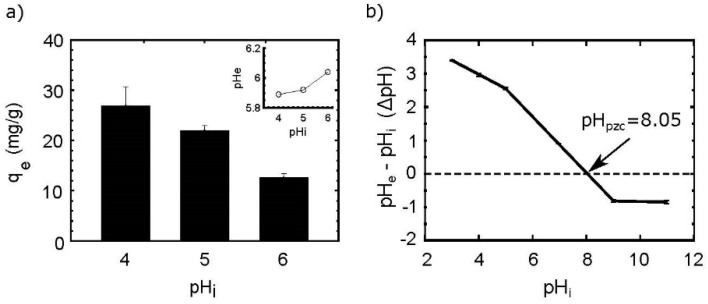
pH dependence of CS-MF. (**a**) pH vs. adsorption uptake, (**b**) pH changes at different pHs in blank solutions. Conditions: (**a**) T: 25 °C, sorbent dosage (SD): 1 g/L; AS: 150 rpm; CT: 24 h; initial concentration (Ci): 50 mg/L; (**b**) T: 25 °C, SD: 1 g/L; AS: 150 rpm; CT: 24 h; electrolyte: NaCl 0.01 M.

The study of pH influence on the q_e_ of Nd(III) was carried out at various pHs of 4, 5, and 6 (Figure 5a). The experiment was executed at these conditions because at pH < 3.5, the beads are dissolved, due to the natural hydrolysis of the CS [39]. The adsorption of Nd(III) by CS-MF beads showed that at pH 4, the major q_e_ was produced, the q_e_ decreased as the pH was increased, and the drop in q_e_ at pH 6 was around 50% compared to that at pH 4.

Figure 5b shows ΔpH (pH_e_ − pH_i_ = ΔpH) in solutions without Nd(III) ions (blank solutions) at different initial pHs. Thus, the ΔpH accounts for the interactions between the CS-MF surface and H^+^ ions, in which a positive ΔpH indicates a binding of H^+^ ions on the CS-MF. The ΔpH shows the largest difference at lower than at higher pHs; therefore, there were more electrostatic attractions at lower pHs, and these decreased as the pH approached the pH_pzc_ (pH_pzc_ = 8.05). In addition, the protonation of amine groups of chitosan under its pK~6 is a well-established phenomenon [40], which provides the conditions for the Nd(III) adsorption. Similarly, comparing the ΔpH produced during Nd(III) adsorption (ΔpH of 1.86 at pH 4) depicted in the inner subfigure in Figure 5a, with the ΔpH at the same pH 4 in Figure 5b (ΔpH of 2.8), it is noted that when Nd(III) ions are present in the solution, they compete with H^+^ ions, and take part of the active surface sites on CS-MF. Various studies presented similar results, which indicates that under pH_pzc_, cation binding takes place [22,24].

On the other hand, it is noticed that the adsorption behavior of MFs and CS-MF along the pH follows an opposite trend, which means that for MFs, higher pHi conditions result in better adsorption capacity (Figure 2a), and for CS-MF, higher pHi represents lower performance (Figure 5a). The better adsorption capacity of the CS-MF composite at lower pH responds to a greater extent to the presence of chitosan, rather than MF-60. We attributed this phenomena to the CS-MS beads composition, which are 66.66% chitosan and 33.33% MF-60-CS:MF in a ratio of 2:1, which, in turn, can be corroborated by the following: (i) the FTIR analysis after Nd(III) adsorption suggests that amino groups of chitosan were the main groups involved in the adsorption (Figure 4a); (ii) at pHi = 4, the CS-MF beads drive stronger electrostatic interactions than MF-60, while the better adsorption performance of MF-60 at pH = 6 is mainly attributed to the physical features (crystallite nanometric size) than to electrostatic interactions (Figure 5b vs. Figure 2b); and (iii) the CS could block some of the active surface sites of MF-60, which can be assumed by the high amorphous pattern presented in the XRD profile of the CS-MF (Figure 4b).

### 3.3. Equilibrium Isotherms

**Figure 6:**
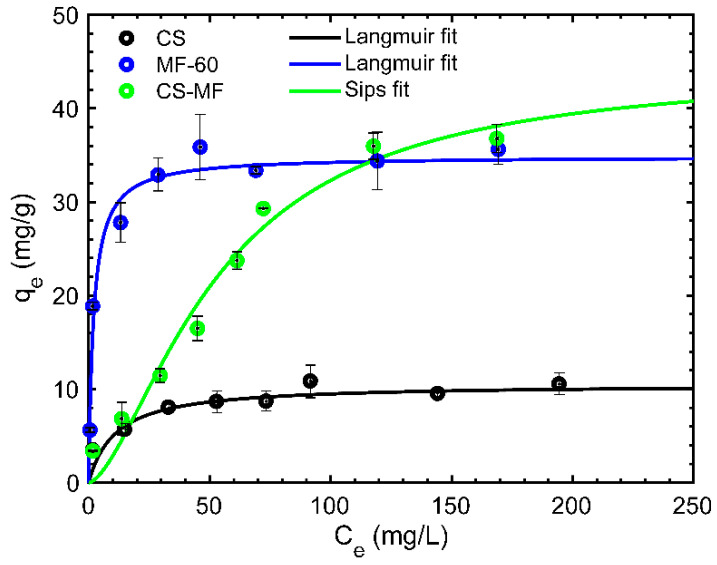
Equilibrium isotherm curves adjusted to Langmuir model (T: 25 °C, SD: 1 g/L; AS: 150 rpm; CT: 24 h; pHi: 4; C_i_: 5–200 mg/L).

The effect of the initial concentration on the adsorption uptake capacity (equilibrium isotherms) is necessary to describe the retention, release, or mobility of a substance from an aqueous system to an adsorbent at constant temperature and pH [41]. Figure 6 shows the impact of the Nd(III) initial concentration in the adsorption process. Isotherm curves of CS-MF, MF-60, and neat CS (raw form) particles were developed to compare the effect of the incorporation of MF-60 in CS.

The Nd(III) adsorption capacity of CS-MF was slightly higher than those of MF-60 and neat CS (CS-MF > MF-60 > CS) at initial Nd concentrations. At C_e_ over 200 mg/L of Nd(III), the CS-MF adsorption capacity was around 5 times higher than that of CS particles (37.28 mg/g for CS-MF vs. 8.20 mg/g for CS), and also showed better adsorption capacity of the MF-60 particles (~34 mg/g).

The experimental data were adjusted to the Langmuir, Freundlich, and Sips models (Table 2). This adjustment is crucial to describe the distribution of the adsorbate between the liquid–solid phases in equilibrium. CS and CF-60 data were adjusted to the Langmuir model with a correlation coefficient (r^2^) of 0.97 and 0.96, respectively, while CS-MF was better adjusted by the Sips model (r^2^ = 0.98). According to the Langmuir theory, adsorption was produced mostly in the monolayer and could be attributed to more homogeneous surfaces, while the Sips theory is related to the more heterogeneous systems [42]. Thus, the incorporation of MF particles into the CS produces a more heterogeneous structure, which is corroborated by SEM observations (Figure 3a,b).

With regard to the sorbate–sorbent affinity, which is related to the “b” Langmuir parameter, it was superior for MF-60 followed by neat CS and CS-MF (Table 2). MF-60 and CS showed a progressive increase in q_e_ until the saturation plateau was reached at C_e_ concentrations close to 30 mg/L; then, the adsorption was limited. For CS-MF, a strict plateau was not observed. The lower affinity of CS-MF was produced by the blocking of Nd(III) ions for the MF particles’ surface; however, as long as the concentration was increased, a higher adsorption capacity was reached, even surpassing the MF-60 q_e_. Thus, the incorporation of MF-60 into CS enhances the adsorption capacity of CS toward Nd(III) ions adsorption, and even improves the q_e_ of the MF-60 particles.

CS-MF beads show competitive performance for Nd(III) recovery. Table 3 presents similar materials with their related sorption capacity. Remarkably, CS-MF was obtained by one of the simplest existing methods. In particular, MF particles were not subjected to the annealing process at high temperatures [9], which, consequently, eliminates the need for large amounts of energy necessary to obtain nano-MF. This study demonstrates that the use of MF particles fixed in CS in the form of beads is suitable for Nd(III) recovery. Additionally, CS-MF beads present some advantages over similar magnetic and non-magnetic materials based on chitosan (Table 3). In terms of q_max_, CS-MF beads are only comparable to the chitosan nano-particles functionalized with diethylenetriamine, authored by [15]. However, the present material is manufactured in the form of beads, which represent an advantage for their industrial application; moreover, the non-chemical modification of CS-MF implies a reduced cost in its obtention.

### 3.4. Kinetics

Kinetic studies are used to determine the contact time required for adsorption between the adsorbate-sorbent, as well as to gain an insight into the accumulation processes [43]. Commonly, PFORE and PSORE are used as simplified models to describe adsorption dynamics, while the Weber and Morris equation is used to evaluate the contribution of the limited film intraparticle diffusion [44].

**Figure 7:**
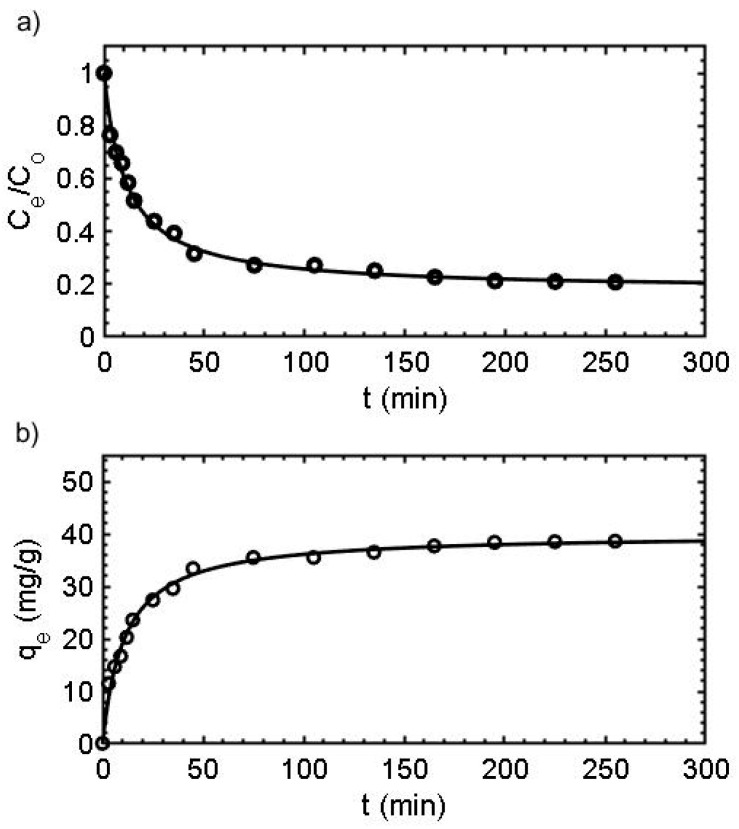
Kinetics isotherms: (**a**) Residual concentration vs. time, (**b**) adsorption capacity vs. time. (Solid line: pseudo-second order (PSORE) fit; Ci 50 mg/L; T: 25 °C, SD: 1 g/L; AS: 150 rpm; CT: 24 h; pHi: 4).

Figure 7 shows the kinetic profile of Nd(III) onto CS-MF, in which three progressive pseudo-steps are observed: An initial step takes around 20 min until reaching a q_e_ of 24.2 mg/g and is attributed to the external diffusion film; a second step, which takes around 75 min, reaches a q_e_ of 32.3 mg/g, related to a mass transfer by pore diffusion throughout the liquid film into the macropores; and a third step, which takes around 180 min, with a q_e_ of 36.5 mg/g corresponding to a surface reaction.

The kinetic data are better adjusted to the FSORE rather than the PFORE model (Table 4). Thus, the occupation rate of Nd(III) ions is of second order regarding the available surface sites [45]. Moreover, PSORE and Elovich suggest chemisorption as the main adsorption mechanism.

On the other hand, the simplified equation brought forward by Weber and Morris evaluated the contribution of the intraparticle diffusion-limited process [44]. The representation of the Weber and Morris equation (Supplementary Figure S2) results in two linear regions, which indicates that the adsorption process is conditioned by two stages. Stage 1 indicates that Nd(III) ions were diffused onto the active sites, and that adsorption was produced at a fast velocity diffusion (Kp_1_) of 5.04 mg/g.min^1/2^. After this, the adsorbate starts the transportation to the inner surface sites and the film diffusion resistance increases. Consequently, the Kp_2_ decreases to 3.04 g/mg*min^1/2^, which also indicates that Nd(III) ions cannot be easily incorporated in this stage.

### 3.5. Reusability

**Figure 8:**
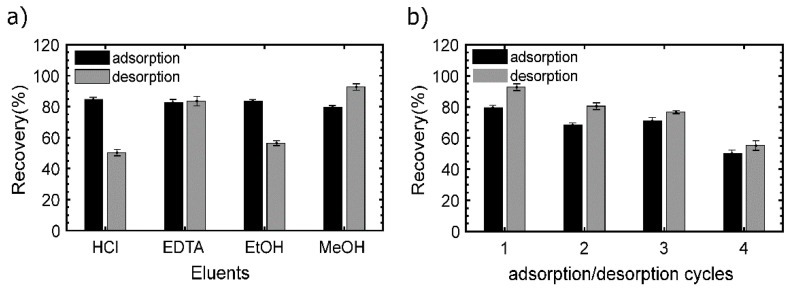
Reusability of the CS-MF beads: (**a**) Screening of the eluents, (**b**) cycles performed by MeOH.

The adsorption–desorption cycles evaluate the reusability of adsorbent materials. In this study, two phases were carried out. Firstly, in one sorption–desorption cycle, with four eluents including HCl and EDTA at pH 10, MeOH and EtOH were tested in order to ascertain the most efficient desorbing solution (Figure 8a). From this first stage, MeOH and EDTA showed better desorption efficiency than the other eluents with up to 80% of desorption, while EtOH and HCl performed 55% and 50%, respectively (Figure 8a). EDTA is considered to be a metal-complexing with high affinity for rare-earth elements, which, in turn, serves as an explanation for its optimal desorption performance, while MeOH has been tested as an excellent desorbing agent of metals from the chitosan matrix.

MeOH was the desorbing agent selected for testing the reusability of the CS-MF, and the material performs up to four adsorption/desorption cycles with efficiencies over 55% (Figure 8b), from which the first three cycles achieve around 80% of recovery; however, in the fourth cycle, both adsorption and desorption efficiency drop to 55%.

## 4. Conclusions

The adsorbent magnetic beads of chitosan-manganese ferrite were developed by the use of a simple procedure, in which the temperature of coprecipitation synthesis of MF was optimized. The hybrid material was successfully applied to Nd(III) recovery from aqueous solutions. The material developed shows acceptable adsorption capacity and equilibrium reaction time; additionally, the material can be reused up to four times and can be removed by the use of a magnetic field.

## Figures and Tables

**Table 1:** Adsorption capacity of different metals with magnetic materials.

Sorbent	pH	q_e_ (mg/g)	Metals	Authors
MnFe_2_O_4_	6	3.39	Pb	[31]
MnFe_2_O_4_	6	25.1	Cr(VI)	[32]
Fe_3_O_4_	8	24.88	Nd(III)	[33]
MF-60	6	37.87	Nd(III)	This work

**Table 2:** Langmuir, Freundlich, and Sips constants of CS-MF, MF-60, and CS.

Material	Langmuir			Freundlich			Sips			
	q_max_ (mg/g)	b (L/mg)	r^2^	K_F_ (mg^1−1/n^/g × L^1/n^)	n	r^2^	q_ms_ (mg/g)	Ks (L/mg)	ms	r^2^
CS-MF	51.69	0.01	0.96	1.95	1.70	0.94	44.29	0.01	0.63	0.97
MF-60	35.85	0.28	0.98	14.9	5.23	0.82	36.73	0.31	1.12	0.97
CS	10.53	0.09	0.97	3.32	4.31	0.95	18.73	0.17	0.40	0.96

**Table 3:** Nd(III) recovery with different materials.

Sorbent	pH	q_max_ (mg/g)	Authors
Cysteine-functionalized chitosan magnetic particles	6	17.1	[16]
Chitosan/iron(III) hydroxide	6	13.8	[22]
Diethylenetriamine-modified magnetic chitosan nanoparticles	7	30.6	[14]
Diethylenetriamine-functionalized chitosan chitosan magnetic nano-based particles	5	50.8	[15]
3-mercaptopropionic acid-tetraethyl orthosilicate ferrite	8	25.58	[17]
CS-MF	4	44.29	This work

**Table 4:** Kinetic parameters of CS-MF.

**Experimental**		**Pseudo-First-Order Rate Equation (PFORE)**			**Pseudo-Second-Order Rate Equation (PSORE)**		
Sorbent		K_1_ (1/min)	q_1_ (mg/g)	r^2^	K_2_ (g/mg × min)	q_2_ (mg/g)	r^2^
CS-MF		0.066	36.69	0.96	0.0023	40.02	0.99
**Elovich Equation**			**Weber and Morris**				
A	β	r^2^	Kp_1_ (g.mg^−1^ × min^−1/2^)	r^2^	Kp_2_ (g/mg × min^1/2^)		r^2^
31.387	0.066	0.97	5.045	0.96	3.045		0.92

## Supplementary Materials

The following are available online at https://www.mdpi.com/2079-4991/10/6/1204/s1, Figure S1: Diagram of chemical species of Nd^3+^, Figure S2: Intraparticle diffusion coefficients using CS-MF as sorbent.
