# Supplementary material for: Recovery of Neodymium (III) from Aqueous Phase by Chitosan-Manganese-Ferrite Magnetic Beads
Source: Nanomaterials (Basel). 2020 Jun 19;10(6):1204. doi: 10.3390/nano10061204 (PMC7353099; doi:10.3390/nano10061204)
Supplement: Supplementary file 1 [file nanomaterials-10-01204-s001.pdf]

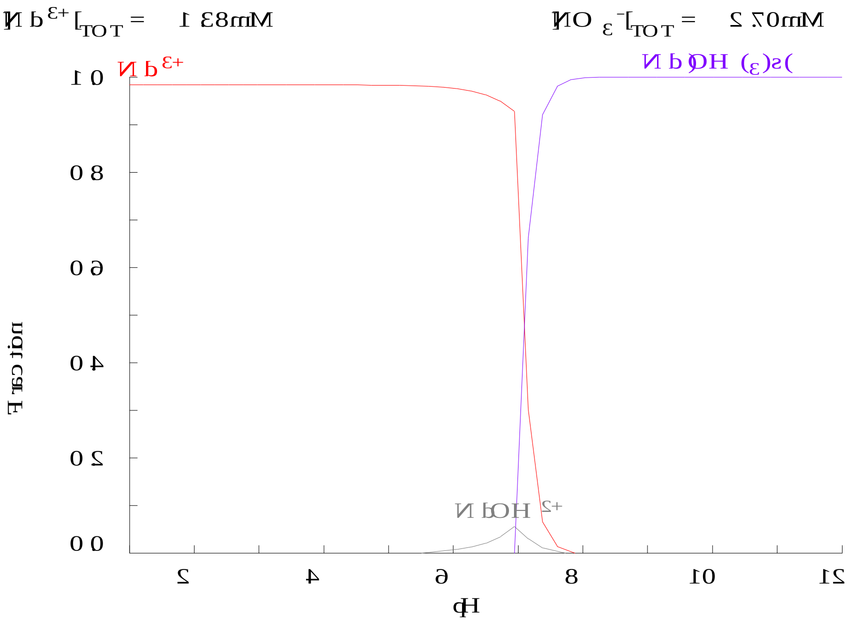

Fig. S1. Diagram of chemical species of Nd^{3+}

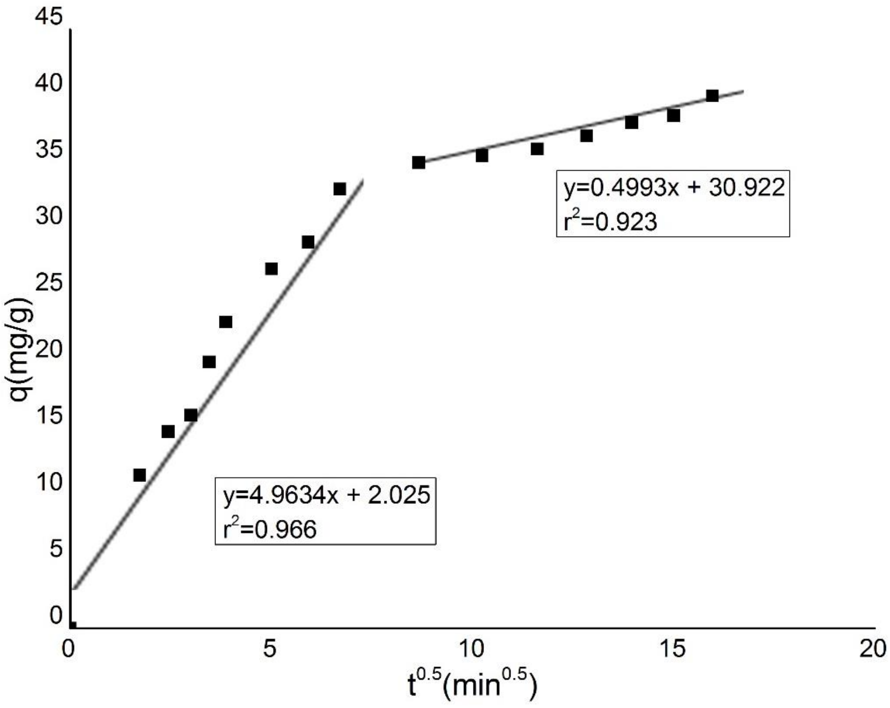

Figure S2. Intraparticle diffusion coefficients using CS-MF as sorbent
